# Impact of Adenovirus E4-ORF3 Oligomerization and Protein Localization on Cellular Gene Expression

**DOI:** 10.3390/v7052428

**Published:** 2015-05-13

**Authors:** Elizabeth I. Vink, Yueting Zheng, Rukhsana Yeasmin, Thomas Stamminger, Laurie T. Krug, Patrick Hearing

**Affiliations:** 1 Department of Molecular Genetics and Microbiology, Stony Brook University, Stony Brook, NY 11794-5222, USA; E-Mails: yueting.zheng@stonybrook.edu (Y.Z.); laurie.krug@stonybrook.edu (L.T.K.); 2 Department of Computer Science, Stony Brook University, Stony Brook, NY 11794, USA; E-Mail: rukhsana.yeasmin@stonybrook.edu; 3 Institute for Clinical and Molecular Virology, University of Erlangen-Nuremberg, Schlossgarten 4, Erlangen 91054, Germany; E-Mail: Thomas.Stamminger@viro.med.uni-erlangen.de

**Keywords:** adenovirus, E4-ORF3, transcription, cellular gene expression

## Abstract

The Adenovirus E4-ORF3 protein facilitates virus replication through the relocalization of cellular proteins into nuclear inclusions termed tracks. This sequestration event disrupts antiviral properties associated with target proteins. Relocalization of Mre11-Rad50-Nbs1 proteins prevents the DNA damage response from inhibiting Ad replication. Relocalization of PML and Daxx impedes the interferon-mediated antiviral response. Several E4-ORF3 targets regulate gene expression, linking E4-ORF3 to transcriptional control. Furthermore, E4-ORF3 was shown to promote the formation of heterochromatin, down-regulating p53-dependent gene expression. Here, we characterize how E4-ORF3 alters cellular gene expression. Using an inducible, E4-ORF3-expressing cell line, we performed microarray experiments to highlight cellular gene expression changes influenced by E4-ORF3 expression, identifying over four hundred target genes. Enrichment analysis of these genes suggests that E4-ORF3 influences factors involved in signal transduction and cellular defense, among others. The expression of mutant E4-ORF3 proteins revealed that nuclear track formation is necessary to induce these expression changes. Through the generation of knockdown cells, we demonstrate that the observed expression changes may be independent of Daxx and TRIM33 suggesting that an additional factor(s) may be responsible. The ability of E4-ORF3 to manipulate cellular gene expression through the sequestration of cellular proteins implicates a novel role for E4-ORF3 in transcriptional regulation.

## 1. Introduction

The outcome of adenovirus (Ad) infection is determined by the interplay between the ability of the host cell to mount an effective antiviral response and the ability of the virus to restrict host cell defenses. Successful Ad replication relies on functions provided by the early region four (E4). This region encodes seven known proteins required for counteracting the host cell antiviral response, effective shutoff of host-cell protein synthesis, late viral mRNA accumulation, and late viral protein synthesis [1,2,3].

The E4-ORF3 and E4-ORF6 proteins have functionally redundant properties sufficient to facilitate virus DNA replication and infectious particle production [1,2]. Together with Ad E1B-55K, E4-ORF6 primarily functions as an adaptor molecule in an E3 cullin-RING ligase complex [4,5], promoting the ubiquitination and proteasome-dependent degradation of substrates, such as p53 [6,7], Mre11-Rad50-Nbs1 (MRN complex proteins) [8], DNA ligase IV [9], integrin α3 [10], and bloom helicase [11]. Rather than targeting proteins for degradation, the 14 kDa E4-ORF3 protein promotes productive Ad infection by oligomerizing into filamentous nuclear inclusions termed tracks [12]. E4-ORF3 nuclear track assembly creates protein binding interfaces and results in the sequestration and inhibition of a variety of cellular proteins [13,14]. This inhibits cellular antiviral properties [15] and serves as a hub for post-translational modifications [16]. Like E4-ORF6, E4-ORF3 targets the MRN DNA repair complex and p53 for inactivation [8,17,18]. Cellular targets unique to E4-ORF3 include PML, TRIM24, and TRIM33 [12,19,20]. Sequestration of cellular proteins into E4-ORF3 nuclear tracks results in inhibition of the DNA damage response, altered p53-mediated signaling, disruption of the interferon-mediated antiviral response, and may influence transcriptional regulation [8,17,18,20,21,22].

Relocalization of MRN complex components Mre11, Rad50, and Nbs1 by E4-ORF3 disrupts activation of the double-strand break repair pathway and may influence cell cycle checkpoint signaling [15]. In the absence of E4 protein products, the MRN complex detects the linear, double-strand Ad genome. The resulting activation of the non-homologous end-joining pathway generates viral genome concatamers, too large to be packaged into the viral capsid [8,17,23,24]. Interestingly, activation of the DNA damage response and concatamer formation is not sufficient to inhibit viral genome replication [25,26]. Associating directly with viral DNA, the MRN complex inhibits genome synthesis by blocking access to the origin of replication [27]. As early as six hours post-infection (hpi), Ad serotype 5 (Ad5) E4-ORF3 sequesters the MRN complex away from virus replication centers and promotes the sumoylation of Mre11 and Nbs1 [8,16,17,25]. The physical removal of MRN proteins is sufficient to allow genome replication and inhibits double-strand break repair [8,17]. Supplementary to E4-ORF3, Ad inhibits the MRN complex as well as downstream non-homologous end-joining repair protein DNA ligase IV through E1B-55K/E4-ORF6-dependent degradation [9].

E4-ORF3 sequesters PML into tracks [12]. As a multifunctional protein, PML has been linked to many different processes through its ability to form punctate structures termed nuclear bodies (PML-NB) [28]. E4-ORF3-dependent relocalization of PML disrupts PML-NB and sequesters PML-NB components Daxx and Sp100. This interferes with the ability of the cell to mount an effective interferon-mediated antiviral response [21,22]. PML-NB protein composition varies between tissue types and cellular conditions; the vast PML interactome implicates PML-NB in a wide variety of cellular processes [28]. PML-NB-associated functions include cell cycle regulation, post-translational modification, DNA damage response, apoptosis, and transcriptional regulation [29]. It remains unclear if relocalization by E4-ORF3 impacts each of these PML-NB-associated functions.

Although E4-ORF3 does not alter p53 localization, E4-ORF3 down-modulates p53-mediated signaling [18]. E4-ORF3 track formation facilitates the colocalization of histone methyltransferases SUV39H1 and SUVH2 at dense cellular DNA; this is thought correlate with the upregulation of heterochromatin production. Forming a continuous scaffold with H3K9me3 heterochromatin at p53 promoter elements, E4-ORF3 indirectly antagonizes the ability of p53 to associate with DNA [18].

The E4-ORF3 protein relocalizes several members of the TRIM protein family into nuclear tracks including TRIM19 (PML), TRIM24 (TIF1α) and TRIM33 (TIF1γ) [12,19,20], although the functional significance of TRIM24 and TRIM33 relocalization remains unclear. TRIM (TRIpartite Motif) family members share conserved N-terminal Ring-B box-Coiled Coil (RBCC) motifs; TRIM24 and TRIM33 contain C-terminal PHD/Bromo domains [30,31,32]. TRIM24 and TRIM33 are multifunctional proteins involved in cellular processes including transcriptional regulation, growth control, regulation of development, and protein post-translational modification [30,31,32]. It is interesting to hypothesize that relocalization of these proteins by E4-ORF3 alters one or more of these properties. The RBCC domains of both TRIM24 and TRIM33 have ubiquitin ligase activity that targets substrates including p53 and Smad4 for ubiquitin-dependent proteasome degradation [33,34]. TRIM33 also directs sumoylation of cellular substrates [35]. TRIM24 acts as a ligand-dependent coregulator of gene expression from nuclear receptor-bound promoter elements, influencing retinoic acid and estrogen-mediated signaling pathways [36,37,38]. Like TRIM24, TRIM33 serves as a multifunctional coregulator of gene expression, influencing TGFβ signaling [38,39]. Functioning as a reader of heterochromatin modifications, the PHD/Bromo domain positions TRIM33 on promoter elements upstream of target genes. This activates the ubiquitin ligase activity of TRIM33 which then alters the composition of regulatory Smad proteins at promoter elements [40,41].

Here, we characterized how E4-ORF3-dependent relocalization of transcriptional regulators alters cellular gene expression. A series of microarray time course experiments spanning 24 hours highlighted over 400 E4-ORF3-regulated genes. By sequentially disrupting E4-ORF3 functions or knocking-down cellular effectors, we determined that these differential expression events rely on nuclear track formation, but that changes in cellular gene expression by E4-ORF3 appear to be independent of MRN and resistant to Daxx and TRIM33 knockdown. These results suggest that an additional factor(s) may be responsible for E4-ORF3 activity. The ability of E4-ORF3 to manipulate cellular gene expression through the sequestration of cellular proteins implicates a novel role for E4-ORF3 in transcriptional regulation.

## 2. Materials and Methods

### 2.1. Cell Culture and Virus Infection

Experiments were carried out in U2OS-Tet (Clontech, Mountain View, CA, USA), U2OS (ATCC, Manassas, VA, USA), and 293FT (Life Technologies, Grand Island, NY, USA) cells. Cells were grown in Dulbecco’s modified eagle medium (DMEM) supplemented with 10% Fetal Clone III (Hyclone, Logan, UT, USA; U2OS-Tet and U2OS cells) or 10% Fetal Bovine Serum (Hyclone; 293FT cells), 2 mM L-glutamine, 100 μM MEM non-essential amino acids, and 1 mM sodium pyruvate. Cells were transfected using Lipofectamine (Life Technologies, Grand Island, NY, USA) or Fugene 6 (Roche, Indianapolis, IN, USA) using the manufacturer’s instruction. Cells were infected with Ad5 E1-replacement viruses that express HA-tagged wild-type or mutant E4-ORF3 proteins for 1 h using 500 particles per cell followed by the addition of fresh medium. E1A replacement viruses that express E4-ORF3 fused to an HA epitope under the control of a CMV promoter include Ad-CMV-HA-ORF3-WT, Ad-CMV-HA-ORF3-N82A, Ad-CMV-HA-ORF3_L103A_, and Ad-CMV-HA-ORF3_D105A/L106A_ [17,26].

### 2.2. Immunofluoresence and Western Blot Analysis

Cells were grown on glass coverslips, transfected, and then infected under the conditions described above. Between 16 and 18 hours post-infection (hpi), cells were washed with PBS, fixed with −20 °C methanol, and blocked for 1 h at room temperature with 10% goat serum diluted into PBS. Primary antibodies were diluted into 10% goat serum block and applied to coverslips for 1 h at room temperature. Coverslips were then washed with PBS and incubated with secondary antibody consisting of tetramethyl rhodamine isothiocyanate (TRITC) labeled anti-rat (Invitrogen) or fluorescein isothiocyanate (FITC) labeled anti-rabbit (Invitrogen) antibody for 30 min at room temperature in the dark. Coverslips were mounted on slides using ImmunoMount (Thermo Shandon, Pittsburgh, PA, USA).

Cell lysates prepared for western blot analysis were resolved on SDS polyacrylamide gels. Proteins were transferred to polyvinylidene fluoride (PVDF) (Hybond-P, GE Healthcare, Pittsburgh, PA, USA) membranes or nitrocellulose membranes (Protran, GE Healthcare, Pittsburgh, PA, USA) overnight at 40 mA. Membranes were blocked for 1 h with 3% BSA in PBS, and then incubated with primary antibody overnight at 4 °C. The membranes were washed and incubated in anti-mouse HRP, anti-rabbit HRP, or anti-rat HRP secondary antibodies for 30 min. Immobilon chemiluminescent HRP substrate (Millipore, Billerica, MA, USA) was used for detection.

Primary antibodies used for immunofluorescence and Western blot analysis include: tubulin (T5192, Sigma Aldrich), PML (PG-M3, sc-966, Santa Cruz Biotechnology, Dallas, TX, USA), Daxx (25C12, Cell Signaling Technology, Danvers, MA, USA), HA peptide (600-401-384, Rockland Immunochemcials, Limerick, PA, USA), rat monoclonal E4-ORF3 (mAb 6A11, [42]), and rabbit polyclonal anti-TRIM33 (generated at Lampire Biological Laboratories, Pipersville, PA, USA).

### 2.3. Creation of an E4-ORF3 Inducible Cell Line

Clontech’s Tet-On system was used to generate a stable E4-ORF3-inducible cell line. A PCR-generated amplicon consisting of the Ad2 E4-ORF3 coding region was cloned into the BamHI and EcoRI restriction enzyme sites of the pUHD10-3 vector, fused at the N-terminus with an HA epitope and placing it under the control of a doxycycline (dox)-inducible promoter element (pTet-HA-E4-ORF3). The resulting plasmid construct was cotransfected into U2OS-Tet-On osteosarcoma cells with pTK-Hyg (Clontech) using Fugene 6. Two days post transfection, cells were treated with 200 μg/mL G418 and 100 μg/mL Hygromycin B to select for cells with pTet-HA-E4-ORF3. Individual, drug-resistant colonies were isolated, amplified, and treated with 1 μg/mL dox for 48 h to screen for HA-E4-ORF3 expression. One cell subclone that exhibited no E4-ORF3 expression minus the addition of dox and levels of E4-ORF3 equivalent to that observed following Ad5 infection was chosen for subsequent use (termed Tet-E4-ORF3 cells).

### 2.4. Microarray Analysis

Three independent aliquots of dox-induced Tet-E4-ORF3 cells were collected at 6, 12, 18, and 24 h time points. Two aliquots of dox-induced parental U2OS-Tet-On cells were collected at 6 and 24 h post induction. Aliquots of untreated Tet-E4-ORF3 and untreated U2OS-Tet-On cells also were collected. Total cellular RNA was isolated from cells using the RNeasy kit (Qiagen, Valencia, CA, USA) according to the manufacturer’s instruction. RNA quality was measured on an Agilent 2100 Bioanalyzer with the RNA 6000 Nano LabChip^®^. Transcripts were prepared with Agilent’s Quick Amp Labeling Kit, two-color, then hybridized to an Agilent 4 × 44k whole human genome expression array. Initial processing of the raw data was performed using Agilent’s Feature Extraction (FE) software v9.5 (Santa Clara, CA, USA). Data were then normalized using the Limma Bioconductor Package (http://www.bioconductor.org/packages/release/bioc/html/limma.html). Results were subject to loess normalization within each array and Aquantile normalization between arrays and then log_2_ transformed. [43,44]. A hierarchical clustering algorithm with centroid linkage implemented by Cluster 3.0 software was used to group expression data from each array replicate and the results were visualized with Java Treeview [45,46].

The data were subject to enrichment analysis via the Database for Annotation, Visualization, and Integrated Discovery (DAVID) [47,48]. Agilent probe names corresponding to genes of interest were uploaded to the database along with a list of probe names corresponding to all genes represented on the microarray as background. Gene Ontology (GO) terms associated with *P*-values less than 0.0001 were considered.

### 2.5. RT-qPCR

Total cellular RNA was isolated from cells using the RNeasy system (Qiagen). 2 μg of RNA were subject to reverse transcription PCR using SSII RT (Invitrogen) according to the manufacturer’s instructions. cDNA pools were diluted 1:10 in dH_2_O. 1 μL of this dilution served as a template for qRT-PCR along with 0.5 μM each forward and reverse primer and 10 μL 2× DyNAmo HS sybr green master mix (Thermo Scientific, Waltham, MA, USA) in a total volume of 20 μL per reaction. Primer pairs were designed with the aid of qPrimerdepot [49] and RTPrimerDB [50] (upplemental Table S4S). Each RT-qPCR run contained two technical replicates of each sample. RT-qPCR was carried out and analyzed on an Applied Biosystem 7500 Real Time PCR System, according to the program: 95 °C for 10 min hot start followed by 40 cycles of 95 °C for 15 s, 60 °C for 1 min. The Pfaffl method of relative quantification was used to convert the resulting threshold cycle data for each sample to relative fold change information [51]. Primer efficiencies were calculated by performing serial dilutions of template cDNA and generating a standard curve. The slope of the standard curve was applied to the equation Efficiency = 10^(−1/slope)^. Efficiencies listed in Supplemental Table 4, column E represent and average value of at least two results.

### 2.6. Generation of Knockdown Cell Lines

Oligonucleotides to express short hairpins RNAs directed against TRIM33 [34] were cloned into the pSIREN-RetroQ vector (Clontech). Constructs containing non-functional shRNA (shControl) or shRNA directed against Daxx (shDaxx) were previously described [52]. 4 μg Retrovirus construct was cotransfected in to 293FT cells along with 4 μg pVSV-G and 4 μg helper virus vector using Lipofectamine 2000. Aliquots of the resulting retrovirus-containing media from transfected cells were collected at 48 h and 72 h post-transfection, clarified by centrifugation, filtered through a 0.45-µm filter, and stored at −80 °C. Undiluted retrovirus stock was added to Tet-E4-ORF3 cells directly or diluted 1:500 into general media before infection in the presence of 5 μg/mL polybrene. Media was replaced after overnight incubation, and the virus was allowed to recombine with host cell genomes. At 48 hpi, cells were treated with 2 μg/mL puromycin to select for stably transfected cells. Pools of cells were screened for knockdown by Western blot analysis for the corresponding protein of interest.

## 3. Results

### 3.1. Creation and Characterization of a Tet-Inducible E4-ORF3 Cell Line

To characterize changes in gene expression that result from E4-ORF3 expression, we generated a stable U2OS-derived cell line that expresses HA-tagged E4-ORF3 in a dox-inducible manner (termed Tet-E4-ORF3 cells). Human U2OS osteosarcoma cells were selected for their ability to express wild-type p53. This approach allows for consistent E4-ORF3 expression while minimizing background transcriptional changes that may have resulted from the use of a viral expression vector. HA-E4-ORF3 was cloned into the pUHD10-3 vector under the control of a Tet response element (TRE). This construct was transfected into U2OS-Tet cells, a cell line that constitutively expresses the reverse tetracycline transactivator protein (rtTA). The introduction of dox allows rtTA to associate with the TRE and induce expression of HA-E4-ORF3. After drug selection, the resulting colonies were screened for their ability to express HA-E4-ORF3 in a dox-inducible manner.

**Figure 1:**
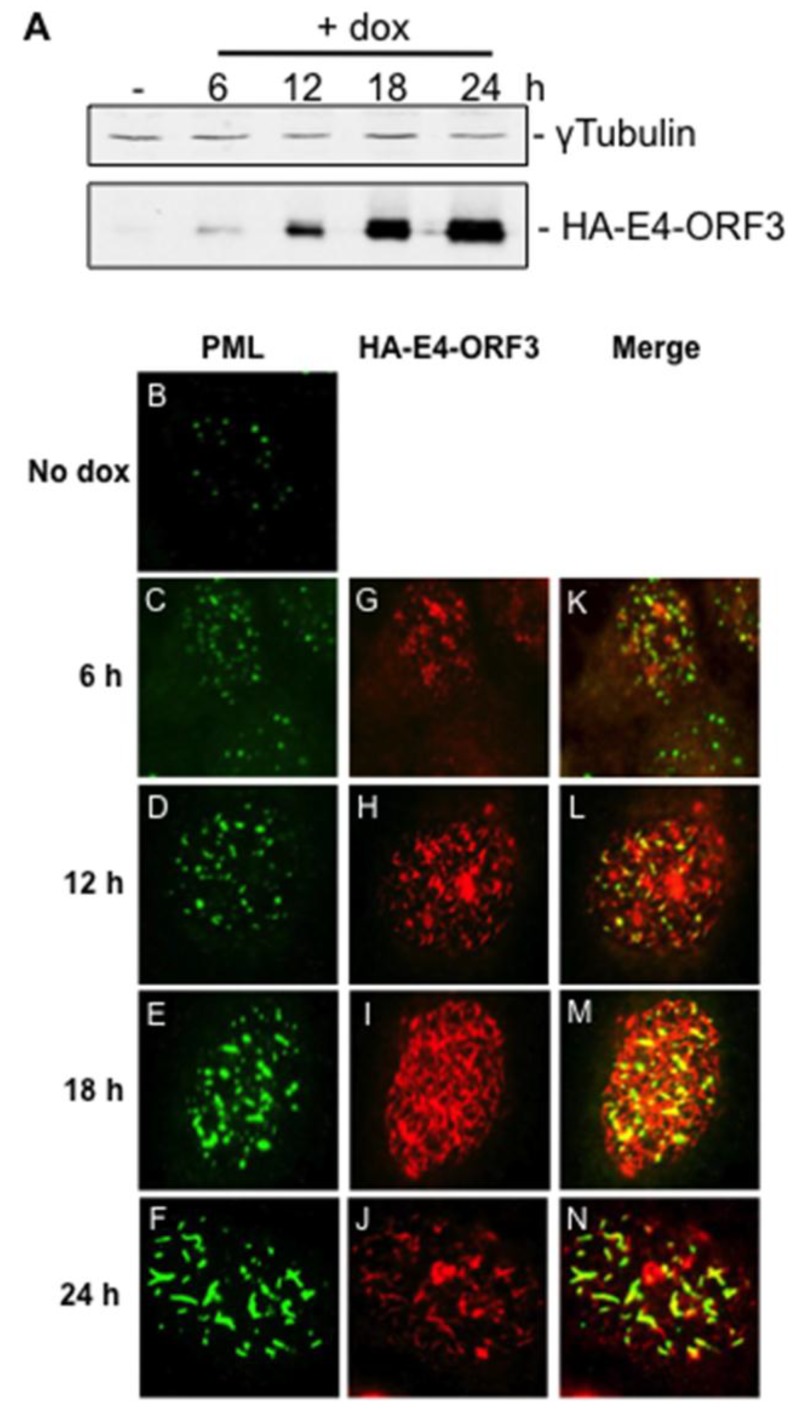
Doxycycline-treated Tet-E4-ORF3 cells produce HA-E4-ORF3 capable of recruiting endogenous PML into nuclear tracks. Tet-E4-ORF3 cells in tissue culture dishes (**A**) or grown on coverslips (**B**–**N**) were mock-treated or supplemented with 1 μg/mL dox for 6, 12, 18, or 24 h. Lysates were subject to Western blot analysis using α-HA antibody or α-γtubulin antibody (A). Cells on coverslips were fixed and stained with an antibody directed against E4-ORF3 or PML followed by TRITC and FITC labeled secondary antibodies. PML localization is shown in (**B**–**F**). HA-E4-ORF3 localization is shown in (**G**–**J**). Merged images are shown in (**K**–**N**).

HA-E4-ORF3 expression in Tet-E4-ORF3 cells was characterized over a 24 h period following dox treatment. Cell lysates were collected at 6, 12, 18, and 24 h post induction (1 μg/mL dox) and analyzed for HA-E4-ORF3 expression by Western blot analysis (Figure 1A). Minimal HA-E4-ORF3 expression was observed in untreated cells. HA-E4-ORF3 accumulation could be detected as early as 6 h post induction, with peak expression at 24 h post-induction. Dox treatment for periods longer than 24 h failed to yield further increases in HA-E4-ORF3 expression. Induced Tet-E4-ORF3 cells express HA-E4-ORF3 to a similar degree and over a similar time frame as E4-ORF3 expression during wild-type Ad infection (Supplementary Figure S1A). To assay for potential cytotoxicity of HA-E4-ORF3 expression or dox treatment, cellular proliferation of dox-treated Tet-E4-ORF3 and parental U2OS-Tet cells was monitored over a period of 96 h. HA-E4-ORF3-expressing cells and untreated Tet-E4-ORF3 cells proliferated to a similar extent for the first 48 h of dox induction (Supplementary Figure 1B). The presence of HA-E4-ORF3 corresponded with impaired proliferation after 48 h. This suggests that a time course experiment spanning 24 h of dox treatment should be free of cytotoxic effects of dox treatment or E4-ORF3 expression.

To confirm the formation of functional E4-ORF3 nuclear tracks, Tet-E4-ORF3 cells were grown on coverslips, stimulated with dox, and analyzed for HA-E4-ORF3 localization and PML reorganization by immunofluoresence (Figure 1). In accordance with the Western blot results, HA-E4-ORF3 tracks could be detected in cells as early as 6-h post induction (G). Over the ensuing 18 h, HA-E4-ORF3 tracks became more elongated and densely packed the nuclei in the majority of cells (H–J). Costaining with an antibody directed against PML revealed that HA-E4-ORF3 efficiently disrupted PML-NB and relocalized PML into nuclear tracks (C–F, merged images K–N). We conclude that Tet-E4-ORF3 cells express functional E4-ORF3 protein in an inducible manner.

### 3.2. Microarray Experiments and Quality Control

Dox-induced HA-E4-ORF3 expression increased over time with maximum detectable protein levels at 24 h post induction (Figure 1). We carried out a time course experiment to observe changes in cellular gene expression over a 24 h period of E4-ORF3 expression. Tet-E4-ORF3 cells were left untreated or were treated with 1 μg/mL dox for 6, 12, 18, and 24 h. Aliquots of cells were taken from each time point and subject to Western blot and immunofluoresence analyses to verify E4-ORF3 expression and track formation. Total cellular RNA was purified from the remaining cells and analyzed by microarray. Transcripts generated from each dox-induced time point were cohybridized to an Agilent 4 × 44k whole human genome expression array along with transcripts generated from an untreated, reference population of Tet-E4-ORF3 cells. This time course experiment was repeated to yield a total of three biological replicates.

To minimize the consideration of genes displaying E4-ORF3-independent changes in expression, we carried out a control microarray experiment. Duplicate batches of parental U2OS-Tet cells were treated with 1 μg/mL dox for 6 or 24 h before RNA purification. The four pools of transcripts were cohybridized to an Agilent 4 × 44k array against the same reference pool of transcripts used in the HA-E4-ORF3 experiment set. Expression changes highlighted in this experiment may represent dox-dependent changes in gene expression, variation resulting from the additional passage of U2OS-Tet cells beyond the creation of the E4-ORF3 expressing cell line, as well as random background expression changes.

Results from both E4-ORF3 time course and control microarrays were normalized using the limma Bioconductor package and were subject to Loess normalization within each array and Aquantile normalization between arrays [43,44]. Gene expression data were determined by comparing transcript levels in the presence of E4-ORF3 or dox relative to transcript levels in the absence of treatment. This was calculated by determining the log_2_ ratio of green to red fluorescent intensity levels. We performed quality control analyses on the resulting data. Fluorescent intensity data from each of the arrays were subject to hierarchical cluster analysis to assess the interrelatedness of each microarray time course replicate (Supplementary Figure 2A). The four control arrays formed a distinct cluster, independent of the E4-ORF3 expression arrays, indicating a high degree of interrelatedness. Arrays from replicates 2 and 3 segregated into early (6 h, 12 h) and late (18 h, 24 h) clusters. Time points produced in replicate 1 formed a distinct cluster in the dendrogram, indicating that a high degree of differential expression in this replicate may be due to technical variation. The distribution of fluorescent intensity across each array was graphed on a density plot (Supplementary Figure S2B). A wider distribution of fluorescent intensities was seen associated with genes on the control arrays than with the test arrays. This suggests that a greater number of genes were differentially expressed under control conditions than in the presence of E4-ORF3. This could indicate that many of the observed changes in transcript levels result from technical, rather than biological variation. To account for any technical variation, we used two methods to select target genes of interest (see below). Identification of a target gene by both methods provides strong evidence of E4-ORF3-dependent expression changes. For one of these methods—selecting genes of interest that display a high degree of differential expression at 24-hours post induction—we only considered data from replicates 2 and 3.

### 3.3. Data Analysis and Target Gene Selection

Two approaches were used to analyze the resulting microarray data cluster analysis and identify genes that show E4-ORF3-dependent differential expression at 24-h post induction relative to 6-h post induction. First, we identified genes with a significant two-fold or greater change in response to E4-ORF3 expression and then subjected that list to gene ontology enrichment analysis. Second, we identified gene with unique E4-ORF3-dependent gene expression profiles by hierarchical clustering. These two approaches selected for genes using different criteria, yielding overlapping, but non-identical pools of target genes.

Determining differential expression at 24-h post induction relative to 6-h post induction selects for genes that share a similar expression pattern as HA-E4-ORF3 (Figure 1). Transcript levels in dox-induced Tet-E4-ORF3 cells were determined relative to transcript levels in dox-treated, parental U2OS-Tet cells to eliminate the inclusion of dox-dependent expression changes. Dox-independent expression changes at 24-h post induction were calculated relative to 6-h post induction to yield a value representing E4-ORF3-dependent expression changes at 24-h post induction relative to 6-h post induction. To search for E4-ORF3-regulated targets, we considered genes that show a two-fold or greater expression change and possess a p-value less than 0.01. 409 genes met these criteria; 289 were up-regulated and 120 were down-regulated at 24-h post induction (Supplementary Table S1).

To look for potential cellular processes influenced by E4-ORF3, we subjected the list of 409 genes to enrichment analysis. The Database for Annotation, Visualization, and Integrated Discovery (DAVID) was used to identify Gene Ontology (GO) terms over-represented in the list of E4-ORF3-regulated targets compared to a background list encompassing all genes represented on the microarray. GO terms associated with *p*-values less than 0.0001 were considered. The 409 E4-ORF3 targets are associated with a variety of terms including cell signaling and cellular defense. This gene list is also enriched in terms implicating growth factor activity and suggests many targets localize to the plasma membrane and extracellular space (Table 1).

**Figure 2:**
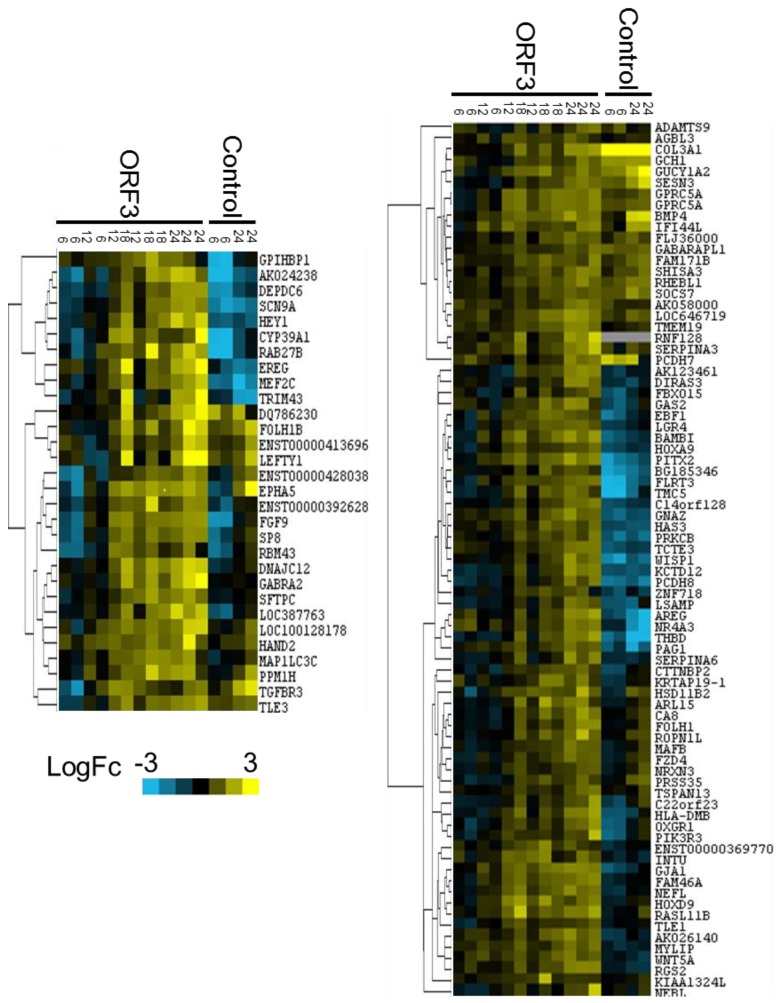
Heat maps and dendrograms corresponding to cluster 1 (**A**) and cluster 2 (**B**).

As a complementary approach to E4-ORF3 target selection, expression change data from each array were subject to hierarchical cluster analysis with centroid linkage using Cluster3.0 [45] then visualized with Java TreeView [46]. Genes that cluster together in the presence of E4-ORF3 but not under control conditions may be coregulated by a common promoter element and/or share functional relevance. Two cluster analyses were carried out. Expression data from 6, 12, 18, and 24 h post-induction time points from Tet-E4-ORF3 replicates 1, 2, and 3 were subject to cluster analysis. Expression data from 6 and 24 h time points from the control, dox-only array were used in a second cluster analysis. Potential clusters of interest were considered on the basis of forming a distinct node in the associated dendrogram and contained genes that display similar expression tendencies within each time point. Finally, genes must not cluster in the absence of E4-ORF3 expression. As most genes did not display differential expression in the presence of E4-ORF3, a high proportion of genes did not fall into distinct, easily discernible clusters. Two clusters were initially considered: cluster 1 contains 31 genes (Figure 2A, Supplementary Table S2) and cluster 2 contains 79 genes (Figure 2B, Supplementary Table S3). Both clusters consist of genes that were either down-regulated or not differentially expressed at 6 h post-induction but were up-regulated after 24 h of E4-ORF3 expression. Gene expression under control conditions failed to match expression patterns seen in the presence of E4-ORF3. Of the 110 genes highlighted by the cluster analysis, 27 also fall into the group of 289 genes up-regulated at 24-h post induction relative to 6-h post induction.

**Table 1:** Enrichment analysis of E4-ORF3 target genes.

Category	Term	Count	%	*p* value
Biological process	Cell surface receptor-linked signal transduction	59	18.97	1.01E-09
	Response to wounding	29	9.32	5.77E-08
	Inflammatory response	19	6.11	8.57E-06
	Defense response	25	8.04	4.95E-05
	Cell-cell signaling	25	8.04	6.65E-05
	Immune response	25	8.04	1.94E-04
	G protein coupled receptor signaling pathway	28	9.00	3.44E-04
	Cell migration	14	4.50	8.35E-04
Cellular component	Extracellular region	74	23.79	2.86E-11
	Extracellular space	36	11.58	1.42E-08
	Integral to plasma membrane	50	16.08	2.03E-08
	Intrinsic to plasma membrane	50	16.08	4.20E-08
	Extracellular region part	42	13.50	1.10E-07
	Plasma membrane part	62	19.93	1.49E-04
Molecular function	Growth factor activity	14	4.50	3.75E-06
	Polysaccharide binding	13	4.18	1.36E-05
	Pattern binding	13	4.18	1.36E-05
	Cytokine activity	14	4.50	2.47E-05
	Glycosoaminoglycan binding	12	3.86	2.86E-05
	Ligand-gated ion channel activity	11	3.54	6.42E-05
	Ligand-gated channel activity	11	3.54	6.42E-05
	Heparin binding	10	3.22	6.47E-05
	Carbohydrate binding	18	5.79	1.00E-04
	Gated channel activity14	16	5.14	2.71E-04

A list of 409 E4-ORF3-regulated genes were subject to enrichment analysis using DAVID. Category indicates the ontology under consideration, whereas the term refers to the specific Gene Ontology (GO) term. Values in the count column specify the number of E4-ORF3-regulated genes that associate with each GO term. The % column indicates the percentage of genes associated with each GO term present in the list of 409 E4-ORF3-regulated genes. The *P* Value column lists modified Fischer Exact *P*-values associated with each enrichment event. Terms with *p*-values less than 0.0001 were considered.

Expression data from E4-ORF3 or control microarray analyses were subject to hierarchical cluster analysis with centroid linkage. Clusters were selected for further analysis on the basis of forming a distinct node unique to the E4-ORF3 dendrogram and maintaining consistent expression patterns within each time point. Blue signal represents down-regulation, whereas yellow indicates up-regulation. Results of E4-ORF3 and control cluster analysis were combined in each heat map for ease of visualization.

### 3.4. Target Gene Validation

E4-ORF3 targets were selected for validation on the basis of displaying a consistently large E4-ORF3-specific change in expression in each biological replicate. Four genes were selected from each cluster of interest: HEY1, TLE3, SP8, and FGF9 from cluster 1 (Figure 2A, Supplementary Table S2) and AREG, BAMBI, PITX2, and RGS2 from cluster 2 (Figure 2B, Supplementary Table S3). Five of these genes—AREG, BAMBI, RGS2, HEY1, and TLE3—were indicated as potential E4-ORF3 targets in both analyses.

**Figure 3:**
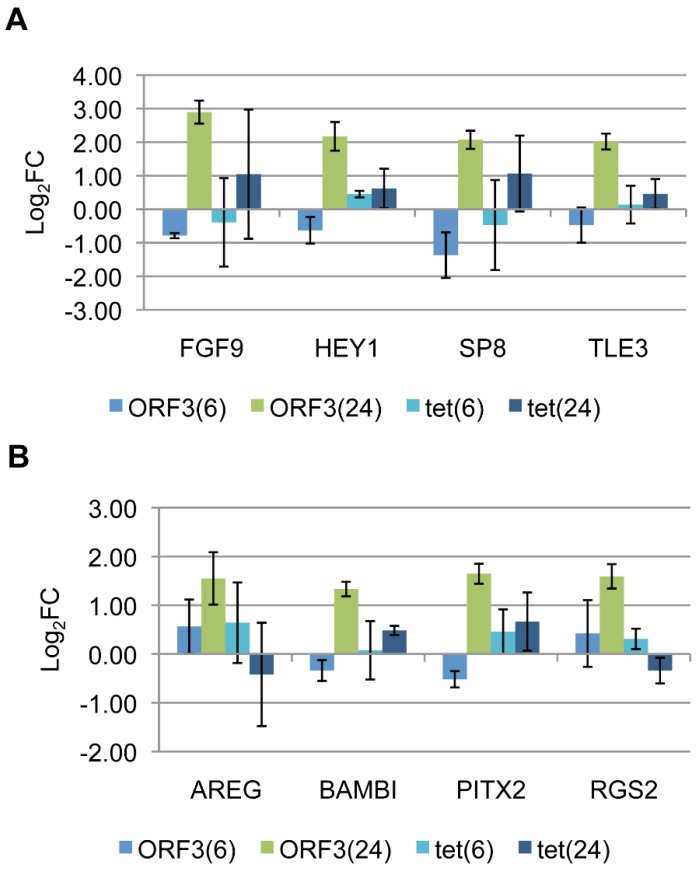
Validation of select E4-ORF3 targets from (**A**) cluster 1 or (**B**) cluster 2 by RT-qPCR with relative quantification. Bars represent Log_2_ fold expression change in dox-treated Tet-E4-ORF3 or U2OS-Tet cells relative to mock treated Tet-E4-ORF3 or U2OS-Tet cells, normalized to GAPDH at 6 and 24 hours post-induction. Error bars = S.D., n = 3.

The ability of E4-ORF3 to influence these eight genes was validated by RT-qPCR (Figure 3). We performed reverse transcription PCR to generate cDNA from mock, 6 h and 24 h RNA pools used in the microarray experiment or from a pool of RNA generated from newly induced Tet-E4-ORF3 cells for a total of three biological replicates. To verify that the expression changes are specific to E4-ORF3, we also generated three sets of cDNA from mock-treated parental U2OS-Tet cells or U2OS-Tet cells treated with 1 μg/mL dox for 6 or 24 h. These batches of cDNA served as templates for qPCR using primers directed against the eight genes of interest. The Pfaffl method of relative quantification was used to calculate changes in gene expression in the presence of dox relative to untreated conditions and normalized to GAPDH [51]. FGF9, HEY1, SP8, and TLE3 demonstrated greater than four-fold E4-ORF3-dependent expression changes by 24-h post induction, although FGF9 and SP8 yielded a two-fold increase in response to dox alone (Figure 3A). AREG, BAMBI, PITX2, and RGS2 displayed greater than a two-fold increase in expression change specifically in the presence of HA-E4-ORF3 by 24-h post induction (Figure 3B).

### 3.5. Linking Differential Gene Expression to E4-ORF3 Functions

The wide array of transcriptional changes induced by E4-ORF3 has the potential to influence many cellular processes. Linking the differential expression of the validated genes to previously characterized E4-ORF3 functions may help to suggest a functional significance for these transcriptional changes in the context of Ad infection. To link differential expression events to E4-ORF3 function, we utilized a series of Ad expression vectors that express wild-type and mutant versions of E4-ORF3. The mutant proteins relocalize a subset of cellular proteins normally targeted by wild-type E4-ORF3 or fail to form tracks entirely.

**Figure 4:**
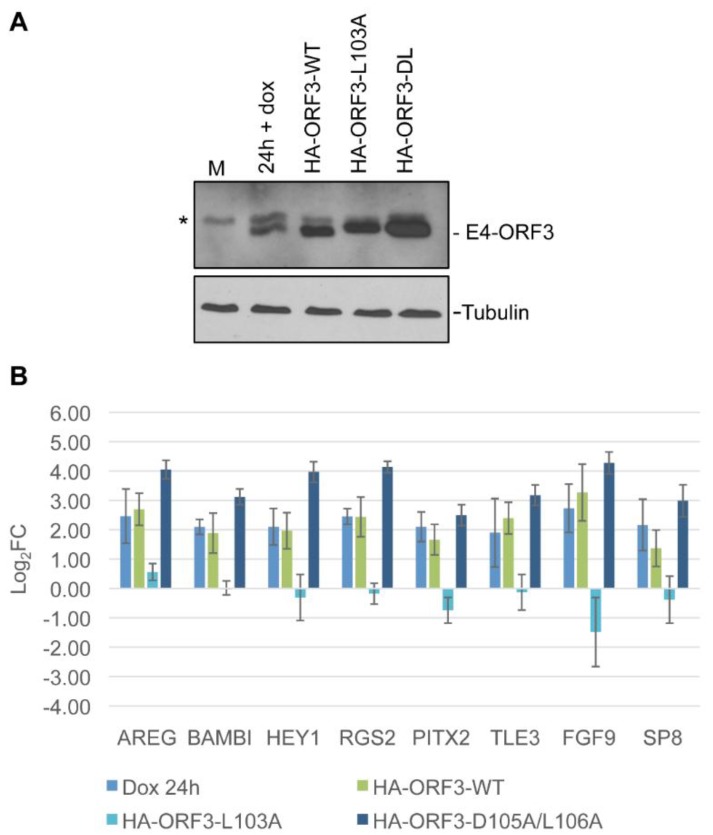
Nuclear track formation, but not sequestration of the MRN complex, is necessary for the up-regulation of select E4-ORF3 target genes. Cells were induced with doxycycline or infected with wild-type or mutant E4-ORF3-expressing Ad vectors to yield an equivalent amount of protein product. E4-ORF3_L103A_ folds properly but fails to form nuclear tracks. E4-ORF3_D105A/L106A_ forms tracks and relocalizes all known targets except the MRN complex. (**A**) Protein levels were verified by Western blot analysis. * Indicates a background band; (**B**) Cellular transcript levels were recorded by relative quantification RT-qPCR. Bars represent Log2 fold change in expression at 24hpi + E4-ORF3, relative to mock, normalized to GAPDH. Error bars = S.D., n = 3. Gene expression differences in the presence of HA-ORF3-WT *vs*. HA-ORF3-L103A are statistically significant (*p* < 0.05) for every target except SP8 as determined by one-way ANOVA analysis with multiple comparisons.

The formation of nuclear tracks serves as a necessary prerequisite for the relocalization of cellular proteins by E4-ORF3. The ability of E4-ORF3 to induce the differential expression of target genes independent of track formation would suggest that E4-ORF3 does not rely on the sequestration of a cellular protein to influence gene expression. This could imply that E4-ORF3 regulates target genes directly, or that HA-E4-ORF3 over-expression induces a cellular stress response independent of E4-ORF3 function. E4-ORF3_L103A_ contains a point mutation in a region necessary for protein oligomerization [13,14]. Although this protein folds properly, it fails to form nuclear tracks. We normalized infection conditions to express mutant or wild-type E4-ORF3 at a level similar to that produced in Tet-E4-ORF3 cells after 24 h of dox treatment (Figure 4A). Tet-E4-ORF3 cells were infected with HA-E4-ORF3_WT_ or HA-E4-ORF3_L103A_ expression viruses for 24 h. As a positive control, Tet-E4-ORF3 cells were treated with 1 μg/mL dox for 24 h. Total cellular RNA was isolated from cells and subject to RT-qPCR. The resulting pool of cDNA served as template with primer pairs directed against the eight validated genes of interest. qPCR results were subject to the Pfaffl method of relative quantification [51]. The experiment was repeated a total of three times (Figure 4B). Whereas wild-type E4-ORF3, either expressed in Tet-E4-ORF3 cells by dox induction or by an Ad expression vector, facilitated the induction of the eight target genes, mutant E4-ORF3_L103A_ failed to induce expression. These results demonstrate that nuclear track formation is necessary for the up-regulation of the eight tested E4-ORF3 target genes and supports a hypothesis that suggests that a cellular protein sequestration event, rather than another E4-ORF3 activity, facilitates differential cellular gene expression. Failure of HA-ORF3_L103A_ to up-regulate the eight genes of interest also suggests that the differential expression events highlighted by the microarray study do not result from a general stress response induced by dox addition and the expression of an ectopic protein.

E4-ORF3 relocalizes proteins of the MRN complex, interfering with the ability of the host cell to detect and repair double-strand DNA breaks. E4-ORF3_D105A/L106A_ contains two point mutations in a region that forms the MRN complex binding interface. This version of E4-ORF3 forms tracks, sequesters PML, TRIM24, and TRIM33, but fails to relocalize the MRN complex [17,26]. To determine if the recruitment of the MRN complex influences the differential expression of the eight validated genes, we mock-infected Tet-E4-ORF3 cells or expressed HA-E4-ORF3_WT_ and HA-E4-ORF3_D105A/L106A_ by virus infection for 24 h (Figure 4A). These cells were used to generate total cellular RNA which served as a template for RT-qPCR, as described above. Both HA-E4-ORF3_WT_ and HA-E4-ORF3_D105/L106_ clearly induced an increase in all target genes (Figure 4B). The increase in the level of induction with the HA-E4-ORF3_D105A/L106A_ mutant protein compared with HA-E4-ORF3_WT_ correlated with increased E4-ORF3 mutant protein expression (Figure 4A). These results suggest that the sequestration of the MRN complex is not necessary for the observed E4-ORF3-dependent up-regulation of cellular gene expression.

**Figure 5:**
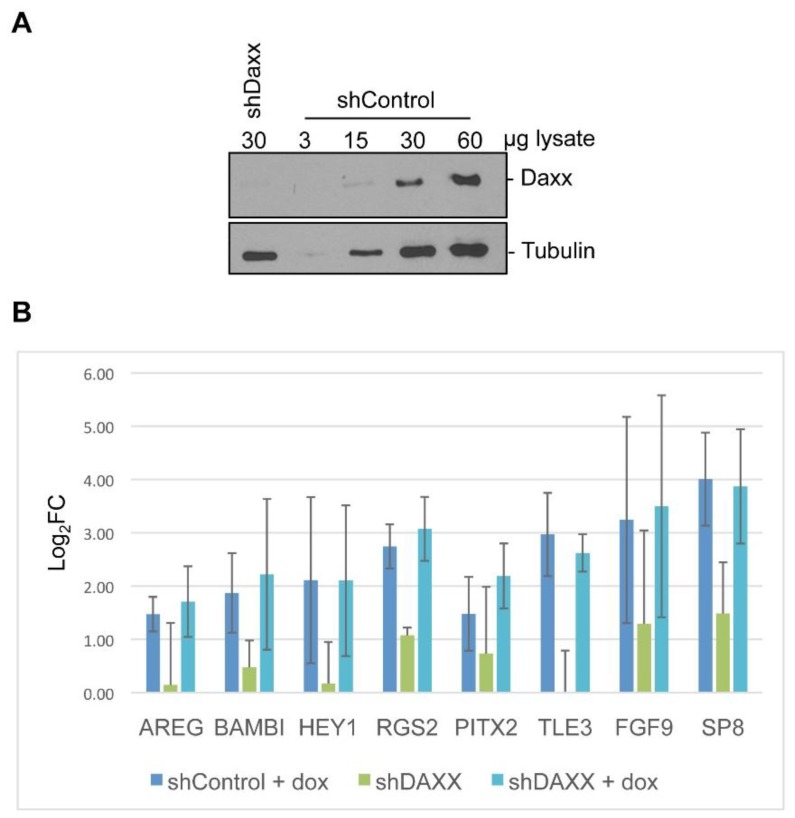
Loss of Daxx does not impede the up-regulation of E4-ORF3 target genes. (**A**) Pools of Tet-E4-ORF3 cells were stably infected with retrovirus that express short hairpin RNA directed against Daxx or a nonspecific control. 60, 30, 15, and 3 μg of shControl lysate were loaded on a polyacrylamide gel with 30 μg of shDaxx and subject to Western blot analysis; (**B**) shDaxx and shControl cells were mock-treated or supplemented with dox to express E4-ORF3 for 24 hours. Transcript levels were recorded by relative quantification RT-qPCR. Bars represent log_2_ fold change in expression in mock treated shDaxx cells, dox-induced shDaxx cells, or dox-induced shControl cells, relative to mock-treated shControl cells, normalized to GAPDH. Error bars = S.D., n = 3. No statistically significant difference in transcript levels could be detected between dox-induced control cells and dox-induced shDaxx cells as determined by one-way ANOVA analysis with multiple comparisons.

**Figure 6:**
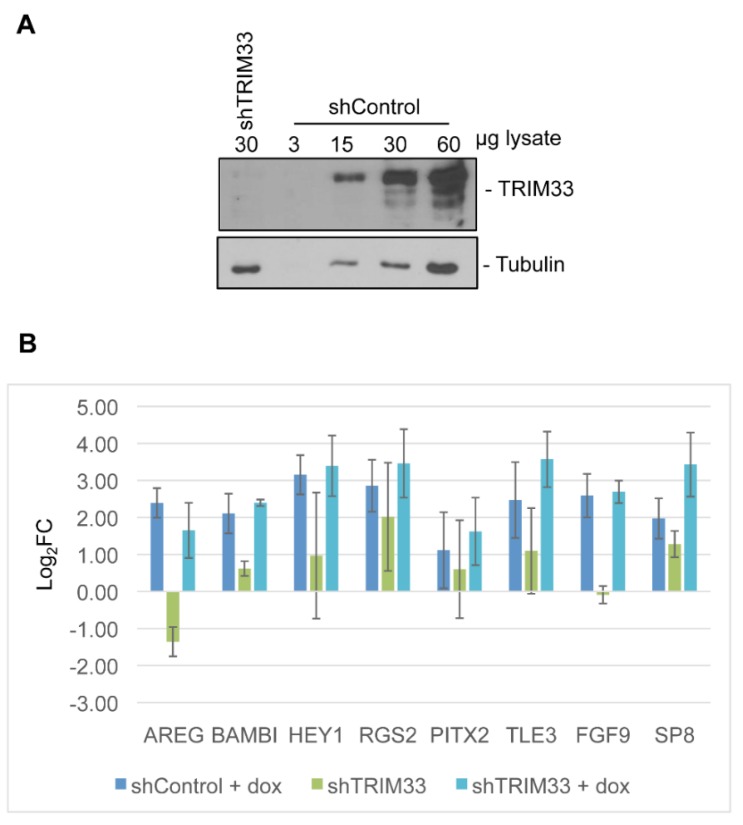
Loss of TRIM33 does not prevent the up-regulation of E4-ORF3 target genes. (**A**) Pools of Tet-E4-ORF3 cells were stably infected with retrovirus that express short hairpin RNA directed against TRIM33 or a nonspecific control. 60, 30, 15, and 3 μg of shControl lysate were loaded on a polyacrylamide gel with 30 μg of shTRIM33 and subject to Western blot analysis; (**B**) shTRIM33 and shControl cells were mock-treated or supplemented with dox to express E4-ORF3 for 24 h. Transcript levels were recorded by relative quantification RT-qPCR. Bars represent log_2_ fold change in expression in mock-treated shTRIM33 cells, dox-induced shTRIM33 cells, or dox-induced shControl cells, relative to mock-treated shControl cells, normalized to GAPDH. Error bars = S.D., n = 3. No statistically significant difference in transcript levels could be detected between dox-induced control cells and dox-induced shTRIM33 cells as determined by one-way ANOVA analysis with multiple comparisons.

Several cellular proteins sequestered into E4-ORF3 nuclear tracks regulate cellular gene expression. Daxx generally represses gene regulation through chromatin remodeling although is may serve as a transcriptional activator in certain cases [53]. TRIM33 serves as a multifunctional coregulator of gene expression, influencing TGFβ signaling and other pathways [38,39]. Daxx and TRIM33 serve as transcriptional effectors that respond to multiple signaling pathways. To examine a potential link between the sequestration of Daxx and TRIM33 with the regulation of cellular gene expression by E4-ORF3, we generated knock-down cell lines in the Tet-E4-ORF3 cell background. shRNAs directed against Daxx or TRIM33, or a non-specific control, were introduced into the inducible E4-ORF3 cell line using retroviruses and reductions in corresponding protein levels was analyzed by Western blot (Figure 5A and Figure 6A). shRNAs against Daxx and TRIM33 knocked-down target protein expression ~5-fold for each protein relative to shControl cells (Figure 5A and Figure 6A).

Control or knock-down cells were left untreated or treated with dox to induce HA-E4-ORF3 expression. At 24-h post induction, total cellular RNA was isolated and used to generate cDNA to be used as a qPCR template. The Pfaffl method of relative quantification was used to determine the transcript levels of the eight genes of interest in knockdown cells relative to uninduced shControl cells (Figure 5B and Figure 6B). Each experimental set was repeated a total of three times. Knockdown of Daxx or TRIM33 did not prevent the E4-ORF3-dependent up-regulation of any of the eight genes analyzed. Knockdown of Daxx alone modestly induced the expression of RGS2, FGF9, and SP8 (Figure 5B). Knockdown of TRIM33 alone reduced AREG expression and induced SP8 and RGS2 expression 2- to 4-fold, respectively (Figure 6B).

## 4. Discussion

Previous studies link E4-ORF3-induced nuclear track formation to disruption of the interferon-mediated antiviral response, inhibition of the DNA damage response, and altered p53-mediated signaling [8,17,18,21,22,25]. Here, we implicate a novel role for E4-ORF3 track formation in the regulation of cellular gene expression. We have identified over four hundred genes that are differentially expressed in response to E4-ORF3 expression, and provide evidence that track formation is necessary for these changes to occur with eight genes that were analyzed further. These E4-ORF3-dependent changes in cellular gene expression appear to be independent of MRN activity and resistant to Daxx and TRIM33 knockdown.

To allow for a more robust examination of potential E4-ORF3 target genes, we utilized two methods of microarray data analysis. First, we considered genes that displayed a two-fold or greater expression change at 24-h post induction relative to 6-h post induction in the presence of E4-ORF3 relative to dox alone (Supplementary Table S1). This method highlights genes that display a high degree of differential expression at times that correspond to high levels of E4-ORF3 expression. As an alternate approach, we performed a cluster analysis (Figure 2, Supplementary Tables S2 and S3). Highlighting genes with similar expression patterns over the time course study, this analysis generates clusters of potentially coregulated genes. These two approaches select for genes using different criteria, yielding overlapping, but non-identical pools of target genes. We considered gene expression trends between 6 and 24 h post induction, as well as the absolute magnitude of differential expression at individual time points. We investigated all genes that met the minimal standards of showing a significant two-fold or greater E4-ORF3-dependent expression change. In addition, we considered genes displaying smaller expression changes that cluster with other potentially relevant E4-ORF3 targets. We believe that using two methods of data analysis allows for a more comprehensive identification of potential genes of interest.

To gain a better understanding of how the E4-ORF3 target genes highlighted by the microarray experiment relate to Ad infection, we subjected the group of 409 E4-ORF3 target genes to gene ontology enrichment analysis (Table 1). Results implicate E4-ORF3 as influencing a wide variety of cellular processes including the regulation of cellular growth properties. Many of the E4-ORF3-influenced genes localize in the extracellular space or in the plasma membrane. It is interesting to speculate that E4-ORF3 influences genes that alter gene expression in neighboring cells, perhaps to prepare them to accept incoming Ad. E4-ORF3-regulated genes also fell into categories related to antiviral effects including inflammatory response, defense response, immune response, and cytokine activity. There was no obvious common transcriptional regulatory mechanism or pathway that relates to the regulation of these diverse genes. The fact that E4-ORF3 up-regulated ~70% of the target genes and down-regulated the remaining ~30% of the target genes (Supplementary Table S1) indicates that E4-ORF3 may influence the activities of different cellular effectors of gene regulation. This idea is consistent with the observation that E4-ORF3 regulates gene expression of some genes early (6-h post induction) and other genes later (by 24-h post induction; Supplementary Tables S2 and S3). The latter observation also is consistent with the possibility that E4-ORF3 influences primary effectors early that result in secondary events at later times after E4-ORF3 expression.

Interestingly, genes that encode E4-ORF3 track-associated proteins were not highlighted by the cluster analysis or through the selection of highly differentially expressed genes at 24-h post induction. PML, MRN components, TRIM24, TRIM33, Daxx, and Sp100 transcript levels remained consistent in the presence or absence of E4-ORF3 expression. This suggests that the cell does not compensate for a decrease in free protein by increasing transcription of these target genes.

E4-ORF3 has been previously shown to inhibit p53-mediated signaling. However, we were unable to detect an enrichment of p53 targets in the population of E4-ORF3-influenced genes. Furthermore, E4-ORF3 did not alter p53 stability or phosphorylation status in Tet-E4-ORF3 cells (data not shown). Stressors including DNA damage, oncogenes, and the Ad E1A protein trigger the activation of p53-mediated signaling [54,55]. P53 likely remains repressed in Tet-E4-ORF3 cells in the absence of these stressors. This may prevent E4-ORF3 from further down-regulating p53-mediated signaling.

Eight genes were validated by RT-qPCR: HEY1, TLE3, SP8, and FGF9 were selected from cluster 1, and AREG, BAMBI, RGS2, and PITX2 were selected from cluster 2 (Figure 2, Supplementary Tables S2 and S3). These targets were selected on the basis of showing a large E4-ORF3-dependent change in expression. Furthermore, these genes of interest correspond to high quality probes on the microarray; displaying high levels of fluorescence relative to background, as well as pixel correlation values approaching one. These genes serve as readout of E4-ORF3-influenced transcriptional regulation. Identifying factors that impact the up-regulation of these genes helps to elucidate how E4-ORF3 may be influencing transcription.

Comparison of transcript levels after the introduction of wild-type or mutant E4-ORF3-expressing Ad vectors revealed that differential expression of the eight validated genes relied on E4-ORF3 nuclear track formation but was independent of the MRN complex. Mutant E4-ORF3_L103A_ folds properly but fails to form nuclear tracks [14]. Failure of this mutant protein to up-regulate target gene expression suggests that the differential expression changes highlighted by the microarray experiment do not result from a stress response induced by an ectopic protein, but likely relies on the sequestration of a cellular protein. Mutant E4-ORF3_D105A/L106A_ targets PML, TRIM24, and TRIM33 for track localization, but fails to sequester the MRN complex [17,26]. Like wild-type E4-ORF3, this mutant protein induced the up-regulation of select target genes. These results indicate that sequestration of the MRN complex does not influence expression of the tested genes, suggesting they function independent of the DNA damage response. To explore the requirement for the track localization of Daxx and TRIM33 on E4-ORF3 transcriptional regulation, we knocked down these proteins in Tet-E4-ORF3 cells. Neither reduction in Daxx nor TRIM33 protein levels prevented the E4-ORF3-dependent up-regulation of any of the eight validated genes. Interestingly, knockdown of Daxx alone modestly induced the expression of RGS2, FGF9, and SP8 (Figure 5B). Knockdown of TRIM33 alone reduced AREG expression and induced SP8 and RGS2 expression 2- to 4-fold, respectively (Figure 6B). TRIM33 and Daxx both have transcriptional repressor properties. It is interesting to speculate that Daxx and TRIM33 contribute to the down-regulation of RGS2, FGF9 and SP8 under normal cellular conditions. Sequestration into E4-ORF3 tracks could inhibit this transcriptional repression. Multiple E4-ORF3 track proteins may influence an overlapping pool of target genes. The simultaneous knockdown of multiple cellular effectors may have a greater influence on transcription than what was observed through knocking down of individual factors.

## 5. Conclusions

We conclude that the Ad E4-ORF3 protein influences the expression of a wide range of cellular genes involved in different cellular functions. E4-ORF3-induced nuclear track formation is necessary to induce these expression changes. The observed expression changes may be independent of Daxx and TRIM33 suggesting that an additional factor(s) may be responsible. The ability of E4-ORF3 to manipulate cellular gene expression through the sequestration of cellular proteins implicates a novel role for E4-ORF3 in transcriptional regulation.

## Supplementary Files

Supplementary File 1
