# Supplementary material for: Impact of Adenovirus E4-ORF3 Oligomerization and Protein Localization on Cellular Gene Expression
Source: Viruses. 2015 May 13;7(5):2428–49. doi: 10.3390/v7052428 (PMC4452913; doi:10.3390/v7052428)
Supplement: Supplementary File 1 [file viruses-07-02428-s001.zip › viruses-82777-supplementary/Supplementary Figure_Table Legends.pdf]

Supplementary Materials

1. Supplementary Figures

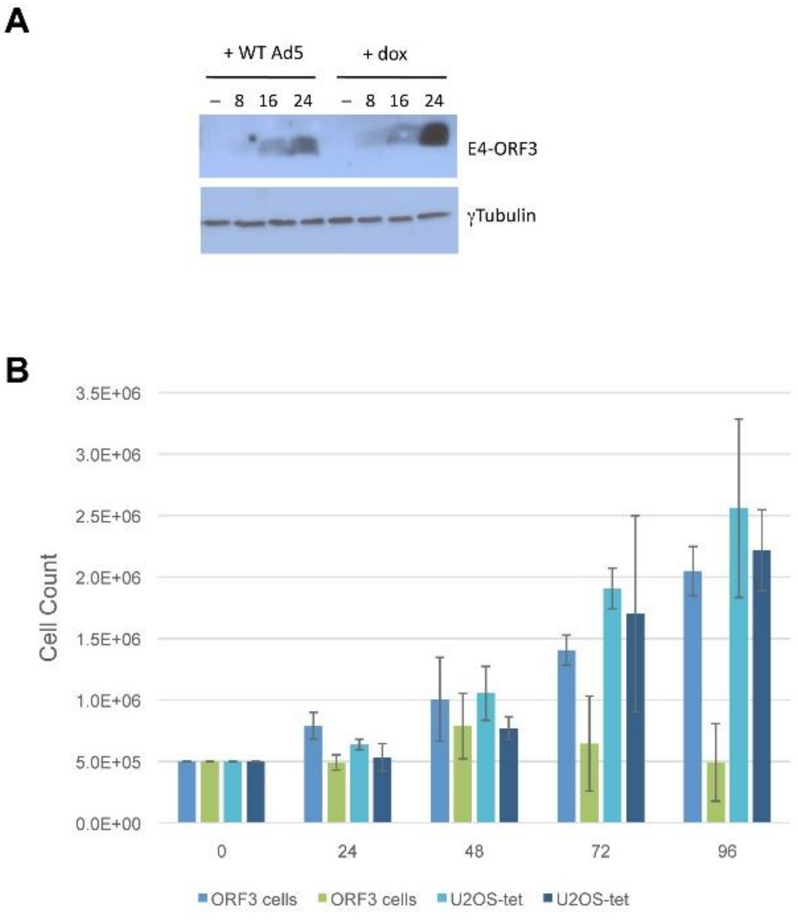

Figure S1. E4-ORF3 expression in Tet-E4-ORF3 cells. **(A)** HeLa cells were uninfected or infected with WT Ad5 and harvested at 8, 16 and 24 hpi (lanes 1-4); Tet-E4-ORF3 cells were uninduced or induced with dox and harvested at 8, 16 or 24 h (lanes 5-8). E4-ORF3 protein levels were analyzed by Western blot. **(B)** Growth kinetics of U2OSTtet and Tet-E4-ORF3 cells in the presence or absence of dox treatment. 5. X 10^5 cells per dish were plated in the presence or absence of 1 µg/ml dox. Cells were counted at 24h intervals. N = 3, error bars = S.D.

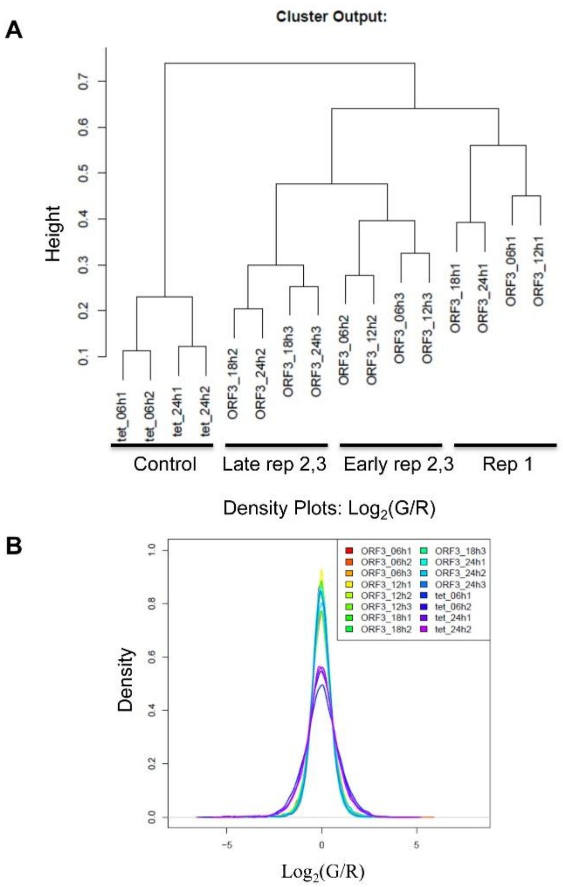

Figure S2. Quality control analysis of microarray data. (**A)** Dendrogram of hierarchical cluster analysis of microarray replicates. **(B)** Density plot of Log_2 ratio of green:red fluorescence (X axis) plotted against percentage of genes possessing that fluorescence ratio (Y axis).

2. Supplementary Tables

Table S1. Genes that display a twofold or greater E4-ORF3-dependent change in expression at 24h post-induction relative to 6h post-induction, $P < 0.01$.

Table S2. Gene expression information corresponding to cluster 1.

Table S3. Gene expression information corresponding to cluster 2.

Table S4. qPCR primer sequences and associated efficiencies. Primer efficiencies were calculated by the equation $E = 10^{(-1/\text{slope of standard curve})}$
